# Synthesis and crystal structure of 3-phenyl-1,4,2-di­thia­zole-5-thione

**DOI:** 10.1107/S205698902200888X

**Published:** 2022-09-13

**Authors:** Melbourne J. Schriver, Tanner George, Jason D. Masuda

**Affiliations:** a Department of Chemistry, Crandall University, PO Box 6004, Moncton, New Brunswick, E1C 9L7, Canada; b Department of Chemistry, Saint Mary’s University, 923 Robie Street, Halifax, Nova Scotia, B3H 3C3, Canada; University of Aberdeen, Scotland

**Keywords:** crystal structure, heterocycle, di­thia­zolone, nitrile sulfide, hydrogen bonding, sulfur inter­actions

## Abstract

In the title compound, C_8_H_5_NS_3_, the dihedral angle between the heterocyclic ring and the phenyl ring is 2.62 (5)°. In the extended structure, aromatic π–π stacking between the 1,4,2-di­thia­zole-5-thione moiety and the phenyl ring is observed [centroid–centroid distances = 3.717 (6) and 3.712 (6) Å]. The almost planar mol­ecules arrange themselves in parallel chains of head-to-tail mol­ecules oriented by a network of weak C—H⋯S contacts close to the sum of their van der Waals radii within the chains. All the hydrogen atoms participate in hydrogen-bonding inter­actions with the sulfur and nitro­gen atoms of adjacent mol­ecules. C=S⋯S contacts between the chains that are significantly shorter than the sum of their van der Waals radii also impact the overall packing.

## Abstract 2

The first crystal structure is reported of a mol­ecule containing the 1,4,2-di­thia­zole-5-thione heterocycle. The packing features aromatic π–π stacking, weak C—H⋯S inter­actions and short S⋯S inter­actions.

## 1. Chemical context

The preparation of derivatives of the 1,4,2-di­thia­zole-5-thione heterocycle was first described in 1967 in 9–14% yield (Behringer & Deichmann, 1967 ▸). Subsequent synthetic work (Greig *et al.*, 1985 ▸) allowed the synthesis of several derivatives in higher yields (21–29%). An investigation of the chemistry of the ring system (Crosby *et al.*, 2002 ▸) showed that the 1,4,2-di­thia­zole-5-thione ring is more thermally stable and less reactive than the electronically similar 1,3,4-oxa­thia­zol-2-one ring but may be used as an alternate route to nitrile sulfides and the thermal cyclo­addition with electron deficient alkynes and nitriles. The existing literature on the 1,4,2-di­thia­zole-5-thione heterocycle is limited to six accounts (Behringer & Deichmann, 1967 ▸; Noel & Vialle, 1967 ▸; Holm & Toubro, 1978 ▸; Greig *et al.*, 1985 ▸; Wai & Sammes, 1990 ▸; Crosby *et al.*, 2002 ▸), which do not include theoretical or crystal-structure determinations.

The 1,4,2-di­thia­zole-5-thione heterocycle is a member of a rich family of isomeric ring systems. Derivatives of 1,2,4-di­thia­zole-5-thione include xanthane hydride, which has been the subject of structural analysis (Stanford, 1963 ▸) and is used industrially as a sulfur-transfer agent in the vulcanization of rubber and the sulfuration of oligonucleotides. The crystal structure of the isomeric ring system 1,3,2-di­thia­zole-4-thione has also been reported (Oakley *et al.*, 1987 ▸).

The incorporation of the preparation, isolation and structural characterization of heterocyclic compounds to demonstrate the chemistry of carb­oxy­lic acid derivatives in the undergraduate organic chemistry laboratory has been previously described (Nason *et al.*, 2017 ▸). To date, our attention has been focused on the synthesis of derivatives of the 1,3,4-oxa­thia­zol-2-one heterocycle because of the relative ease of preparation and, until recently, the limited research studying the chemistry of the heterocycle family. In our search for a new focus heterocycle, the small library of existing publications on the 1,4,2-di­thia­zole-5-thione derivatives coupled to the relative ease of synthesis made this ring system a target for investigation and we now describe the synthesis and crystal structure of the title compound, C_8_H_5_NS_3_. 
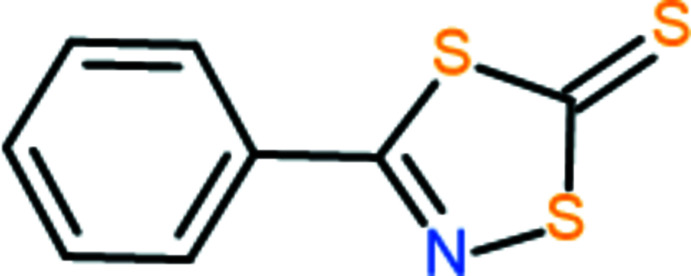


## 2. Structural commentary

**Figure 1:**
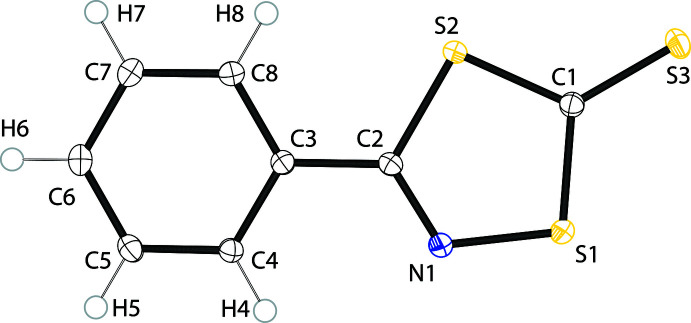
The mol­ecular structure of the title compound, showing anisotropic displacement ellipsoids projected at 50% probability.

The structure of the title compound (Fig. 1 ▸) reveals that the heterocycle and the aromatic ring are essentially co-planar [C8—C3—C2—S2 = −2.91 (13)°]. The C2—C3 [1.4721 (14) Å] bond is not significantly shorter than the accepted value for a C*sp* ^2^—C*sp* ^2^ single bond (1.48 Å) but it is longer than the average (1.45 ± 0.03 Å) of similar C*sp* ^2^—C*sp* ^2^ inter-ring bonds found in the related oxa­thia­zolone derivatives (Nason *et al.*, 2017 ▸). The extension of π delocalization between the rings is sufficient to direct the observed co-planarity. The sum of the inter­nal angles of the heterocyclic ring (539.9°) is almost ideal for five membered rings (540°).

Within the heterocycle moiety, the mol­ecule shows significant (*p* < 0.01) structural differences (Kooijman, 2005 ▸) to similar regions in the related oxa­thia­zolone derivatives, and for reference, the comparison will be made to 5-phenyl-1,3,4-oxa­thia­zol-2-one (Schriver *et al.*, 1995 ▸). In the title compound, the C2=N1 double bond [1.2961 (13) Å] is significantly longer (and weaker) than in the oxa­thia­zolone [1.268 (6) Å] while the C1—S1 bond [1.7248 (11) Å] is shorter [1.754 (5) Å]. These differences are consistent with a higher degree of π delocalization in the title heterocycle as compared to the oxa­thia­zolone. The current π-island structural model for oxa­thia­zolone heterocycles has been suggested to explain the deca­rboxylation to form the nitrile sulfides (Krayushkin *et al.*, 2010 ▸) with longer, weaker endocyclic C—S bonds consistent with lower extrusion temperatures (Zhu *et al.*, 2017 ▸). Conversely, in the title mol­ecule the C1—S1 bond is shorter and stronger, which is consistent with the observed resistance of 1,4,2-di­thia­zole-5-thio­nes to thermally extrude CS_2_ to form nitrile sulfides (Greig *et al.*, 1985 ▸). The endocyclic C—S bonds are significantly (*p* < 0.01) asymmetric with the C1—S1 bond the shortest of the three bonds, consistent with a higher bond order and π character while the C1—S2 bond [1.7363 (11) Å] is longer but not as long as the C2—S2 bond [1.7587 (10) Å]. This pattern of bond lengths is in agreement with a more extensive, and less localized, π delocalization in this heterocycle than in the comparable oxa­thia­zolone derivatives.

Comparison of the structure of the title compound with the structures of the isomeric ring systems 1,2,4-di­thia­zole-5-thione (Stanford, 1963 ▸), 1,3,2-di­thia­zole-4-thione (Oakley *et al.*, 1987 ▸), 1,2,3-di­thia­zole-5-thione (Constanti­nides *et al.*, 2021 ▸) and the derivatives of 1,4,2-di­thia­zole (Oakley *et al.*, 1993 ▸) reveal that the endocyclic C—S bonds in the heterocycle (average 1.74 ± 0.02 Å) and the exocyclic C1—S3 bond [1.6438 (11) Å] are all consistent with the distances expected based on the conventional Lewis structure and the statistical averages for comparable bond distances (C—S = 1.75 ±0.02 Å and C=S = 1.64 ± 0.02 Å) from the comparison heterocycle systems.

## 3. Supra­molecular features

**Figure 2:**
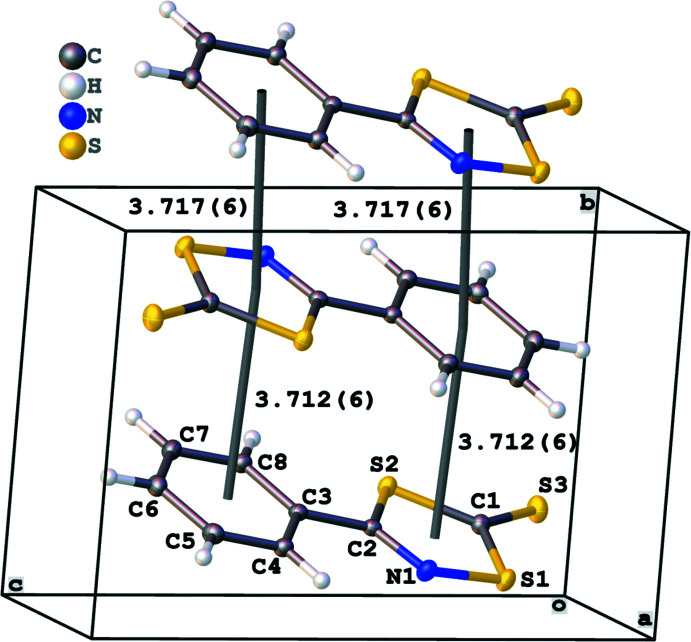
Packing diagram illustrating centroid-stacking inter­actions down the *b-*axis direction (*a*) between parallel-aligned ring systems with co-planar mol­ecules flipped across an inversion centre [centroid-to-centroid distance = 3.712 (6) Å] and packed back to back [centroid-to-centroid distance = 3.717 (6) Å]. The three mol­ecules are staggered with an angle of 166.698 (17)° and two mol­ecules fit within the the *P* 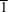 unit cell.

**Figure 3:**
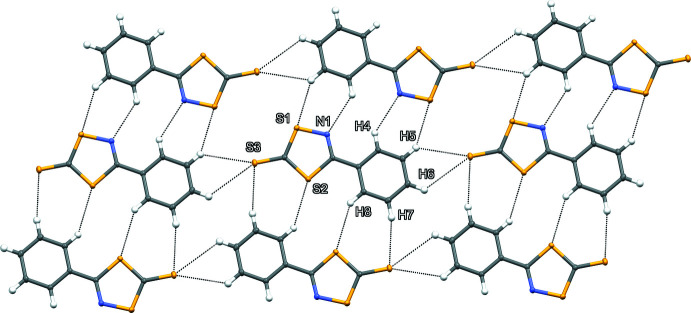
A packing diagram of the title compound showing hydrogen bonding in head-to-tail chains with flanking inter­actions where all possible hydrogen-bond donors and acceptors are participating in hydrogen bonds.

**Figure 4:**
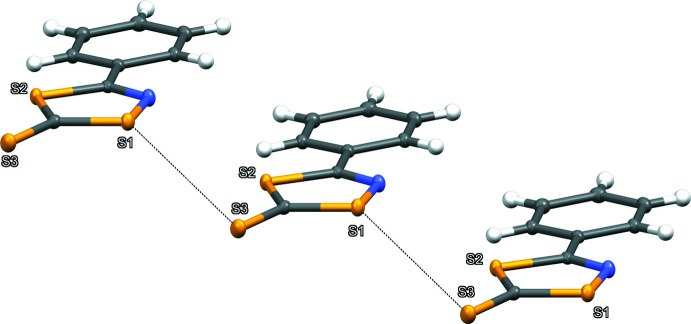
A packing diagram of the title compound showing inter­chain S⋯S contacts of 3.575 (11) Å and the stepwise progression of the chains going down the *b-*axis, stepping towards the *a-*axis.

The extended structure of the title compound features π–π centroid stacking (Fig. 2 ▸), six hydrogen-bonding inter­actions (Table 1 ▸; Fig. 3 ▸) and one chain of sulfur–sulfur inter­actions (Fig. 4 ▸). The packing of two mol­ecules across an inversion centre results in one of two centroid-stacking inter­actions. The mol­ecules exists as co-planar and parallel chains of heterocycles, with supra­molecular contacts confirmed by the statistically constant centroid-to-centroid distances between rings in different adjacent chains [3.717 (6) and 3.712 (6) Å] with the latter centroid-to-centroid distance across the inversion centre. The plane of the mol­ecule is roughly perpendicular to the *b* axis and the mol­ecular centroids form a chain-to-chain, stepwise angle [166.698 (17) °] on the *a* axis. Head-to-toe hydrogen-bonding inter­actions between C5 and C6 donors to the exocyclic thione S3 with H⋯S distances of 2.96 and 3.11 Å, respectively (Fig. 3 ▸) form the primary cohesion along the *a*- and *c*-axis directions. In addition, the rest of the phenyl ring hydrogen atoms are involved with side-on, out-of-plane step-wise hydrogen bonds between H4 and N1 (2.89 Å), H5⋯S1 (3.04 Å), H7⋯S3 (3.06 Å) and H8⋯S2 (3.10 Å). The sulfur–sulfur inter­actions occur as a chain out of plane between the thione S3 and S1 atoms within the ring (Fig. 4 ▸). While the observed H⋯S hydrogen bonding between the mol­ecules is weak (Σ van der Waals radii S⋯H = 3.0 Å), they aid the orientation of the mol­ecules within the out-of-plane chains. In contrast, the S⋯S contact distance [3.575 (11) Å] may appear to be close to the accepted sum of the van der Waals radii (3.6 Å) but when the known anisotropy of sulfur contacts [in plane S⋯S contact 3.20 Å and perpendicular S⋯S contact 4.06 Å (Constanti­nides *et al.*, 2021 ▸)] are factored, it is revealed that the contact is a significant contributor to the supra­molecular packing of the compound.

## 4. Database survey

A search of the Cambridge Structural Database (Version 5.41, September 2021; Groom *et al.*, 2016 ▸) revealed that there are six crystal structures reported for mol­ecules containing the neutral 1,4,2-di­thia­zole heterocyclic ring (Chu *et al.*, 1993 ▸; Oakley *et al.*, 1993 ▸, 1994 ▸; Feng *et al.*, 2016 ▸). The thione moiety in the structure of 3-phenyl-1,4,2-di­thia­zole-5-thione, however, makes this the first crystal structure reported for this heterocyclic system with a thione substituent at the C1 position.

## 5. Synthesis and crystallization

**Figure 5:**
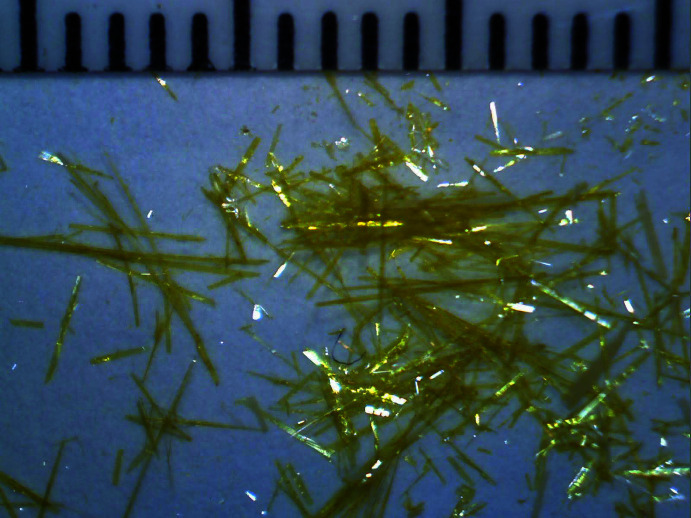
A photograph of crystals of the title compound (1 mm reference scale).

A solution of tri­chloro­methane­sulfenyl chloride (10.17 g, 22.81 mmol) in chloro­form (10.17 g) was added dropwise to a warmed solution of thio­benzamide (6.161 g, 44.91 mmol) in chloro­form (240 ml) according to a literature procedure (Greig *et al.*, 1985 ▸). The reaction mixture was refluxed for 4 h followed by evaporation in a crystallizing dish to a yellow–orange residue (7.002 g). The crude product was recrystallized twice in 95% ethanol to give the product as bright-yellow crystalline needles (Fig. 5 ▸) (1.235 g, 5.84 mmol, 13.0%) suitable for crystallographic analysis. *R*_f_ (CH_2_Cl_2_) = 0.671; UV–visible (CH_2_Cl_2_) λ_max_ nm (log ɛ): 256 (4.29), 361 (4.20), ^1^H NMR 60 MHz, CDCl_3_) δ = 7.53 ppm (multiplet).

## 6. Refinement

Crystal data, data collection and structure refinement details are summarized in Table 2 ▸. All H atoms were geometrically placed (C—H = 0.95 Å) and refined as riding atoms with *U*_iso_(H) = 1.2*U*_eq_(C).

## Supplementary Material

Crystal structure: contains datablock(s) I. DOI: 10.1107/S205698902200888X/hb8037sup1.cif

Structure factors: contains datablock(s) I. DOI: 10.1107/S205698902200888X/hb8037Isup2.hkl

CCDC reference: 2205416

Additional supporting information:  crystallographic information; 3D view; checkCIF report

## Figures and Tables

**Table 1:** Hydrogen-bond geometry (Å, °)

*D*—H⋯*A*	*D*—H	H⋯*A*	*D*⋯*A*	*D*—H⋯*A*
C4—H4⋯N1^i^	0.95	2.89	3.6281 (14)	136
C5—H5⋯S1^i^	0.95	3.04	3.8474 (11)	144
C5—H5⋯S3^ii^	0.95	2.96	3.6529 (11)	131
C6—H6⋯S3^ii^	0.95	3.11	3.7231 (12)	124
C7—H7⋯S3^iii^	0.95	3.06	3.9651 (12)	159
C8—H8⋯S2^iii^	0.95	3.10	3.9141 (11)	145

Symmetry codes: (i) 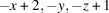 ; (ii) 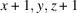 ; (iii) 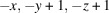 .

**Table 2:** Experimental details

Crystal data	
Chemical formula	C_8_H_5_NS_3_
*M*_r_	211.31
Crystal system, space group	Triclinic, *P* 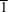
Temperature (K)	100
*a*, *b*, *c* (Å)	5.7955 (4), 7.3789 (5), 10.0344 (7)
α, β, γ (°)	89.459 (3), 89.719 (2), 78.956 (2)
*V* (Å^3^)	421.15 (5)
*Z*	2
Radiation type	Mo *K*α
μ (mm^−1^)	0.81
Crystal size (mm)	0.2 × 0.12 × 0.05

Data collection	
Diffractometer	Bruker APEXII CCD
Absorption correction	Multi-scan (*SADABS*; Krause *et al.*, 2015 ▸)
*T*_min_, *T*_max_	0.654, 0.748
No. of measured, independent and observed [*I* > 2σ(*I*)] reflections	49509, 4085, 3322
*R*_int_	0.064
(sin θ/λ)_max_ (Å^−1^)	0.833

Refinement	
*R*[*F* ^2^ > 2σ(*F* ^2^)], *wR*(*F* ^2^), *S*	0.031, 0.074, 1.03
No. of reflections	4085
No. of parameters	109
H-atom treatment	H-atom parameters constrained
Δρ_max_, Δρ_min_ (e Å^−3^)	0.63, −0.39

Computer programs: *APEX4* and *SAINT* (Bruker, 2019 ▸), *SHELXT2014/5* (Sheldrick, 2015*a* ▸), *SHELXL2018/3* (Sheldrick, 2015*b* ▸) and *OLEX2* (Dolomanov *et al.*, 2009 ▸).

## supplementary crystallographic information

### Crystal data

**Table:**

C_8_H_5_NS_3_	*Z* = 2
*M**_r_* = 211.31	*F*(000) = 216
Triclinic, *P*1	*D*_x_ = 1.666 Mg m^−^^3^
*a* = 5.7955 (4) Å	Mo *K*α radiation, λ = 0.71073 Å
*b* = 7.3789 (5) Å	Cell parameters from 9861 reflections
*c* = 10.0344 (7) Å	θ = 2.8–39.7°
α = 89.459 (3)°	µ = 0.81 mm^−^^1^
β = 89.719 (2)°	*T* = 100 K
γ = 78.956 (2)°	Needle, yellow
*V* = 421.15 (5) Å^3^	0.2 × 0.12 × 0.05 mm

### Data collection

**Table:**

Bruker APEXII CCD diffractometer	3322 reflections with *I* > 2σ(*I*)
φ and ω scans	*R*_int_ = 0.064
Absorption correction: multi-scan (SADABS; Krause *et al.*, 2015)	θ_max_ = 36.3°, θ_min_ = 2.0°
*T*_min_ = 0.654, *T*_max_ = 0.748	*h* = −9→9
49509 measured reflections	*k* = −12→12
4085 independent reflections	*l* = −16→16

### Refinement

**Table:**

Refinement on *F*^2^	Primary atom site location: dual
Least-squares matrix: full	Hydrogen site location: inferred from neighbouring sites
*R*[*F*^2^ > 2σ(*F*^2^)] = 0.031	H-atom parameters constrained
*wR*(*F*^2^) = 0.074	*w* = 1/[σ^2^(*F*_o_^2^) + (0.0293*P*)^2^ + 0.2221*P*] where *P* = (*F*_o_^2^ + 2*F*_c_^2^)/3
*S* = 1.03	(Δ/σ)_max_ = 0.001
4085 reflections	Δρ_max_ = 0.63 e Å^−^^3^
109 parameters	Δρ_min_ = −0.39 e Å^−^^3^
0 restraints	

### Special details

**Table:**

Geometry. All esds (except the esd in the dihedral angle between two l.s. planes) are estimated using the full covariance matrix. The cell esds are taken into account individually in the estimation of esds in distances, angles and torsion angles; correlations between esds in cell parameters are only used when they are defined by crystal symmetry. An approximate (isotropic) treatment of cell esds is used for estimating esds involving l.s. planes.

### Fractional atomic coordinates and isotropic or equivalent isotropic displacement parameters (Å^2^)

**Table:**

	*x*	*y*	*z*	*U*_iso_*/*U*_eq_	
S1	0.59859 (5)	0.10273 (4)	0.20602 (3)	0.01367 (6)	
S2	0.23050 (4)	0.29112 (4)	0.36958 (3)	0.01254 (6)	
S3	0.12785 (5)	0.22623 (4)	0.08266 (3)	0.01711 (6)	
N1	0.67826 (16)	0.13417 (13)	0.36131 (9)	0.01333 (16)	
C1	0.30604 (19)	0.20788 (15)	0.21084 (10)	0.01200 (17)	
C2	0.51316 (18)	0.22144 (14)	0.43743 (10)	0.01050 (16)	
C3	0.55288 (18)	0.26295 (14)	0.57773 (10)	0.01094 (16)	
C4	0.77430 (18)	0.20050 (14)	0.63514 (10)	0.01211 (17)	
H4	0.898918	0.133069	0.583378	0.015*	
C5	0.81060 (19)	0.23770 (15)	0.76809 (11)	0.01428 (18)	
H5	0.960741	0.195896	0.806938	0.017*	
C6	0.6283 (2)	0.33594 (15)	0.84490 (11)	0.01488 (18)	
H6	0.653943	0.359485	0.936024	0.018*	
C7	0.4088 (2)	0.39949 (15)	0.78795 (11)	0.01443 (18)	
H7	0.284614	0.467081	0.839959	0.017*	
C8	0.37177 (19)	0.36371 (14)	0.65470 (10)	0.01259 (17)	
H8	0.222345	0.408062	0.615693	0.015*	

### Atomic displacement parameters (Å^2^)

**Table:**

	*U*^11^	*U*^22^	*U*^33^	*U*^12^	*U*^13^	*U*^23^
S1	0.01186 (11)	0.01664 (12)	0.01185 (11)	−0.00088 (9)	0.00020 (8)	−0.00335 (8)
S2	0.00949 (10)	0.01580 (12)	0.01156 (10)	−0.00040 (8)	−0.00086 (8)	−0.00118 (8)
S3	0.01444 (12)	0.02474 (14)	0.01221 (11)	−0.00380 (10)	−0.00353 (9)	0.00023 (9)
N1	0.0115 (4)	0.0153 (4)	0.0124 (4)	−0.0004 (3)	−0.0012 (3)	−0.0028 (3)
C1	0.0116 (4)	0.0132 (4)	0.0115 (4)	−0.0033 (3)	0.0001 (3)	−0.0004 (3)
C2	0.0103 (4)	0.0095 (4)	0.0118 (4)	−0.0020 (3)	−0.0009 (3)	0.0005 (3)
C3	0.0116 (4)	0.0096 (4)	0.0116 (4)	−0.0020 (3)	−0.0006 (3)	−0.0001 (3)
C4	0.0116 (4)	0.0119 (4)	0.0124 (4)	−0.0013 (3)	−0.0008 (3)	−0.0007 (3)
C5	0.0143 (4)	0.0142 (4)	0.0142 (4)	−0.0025 (4)	−0.0036 (3)	0.0005 (3)
C6	0.0188 (5)	0.0147 (4)	0.0118 (4)	−0.0047 (4)	−0.0013 (4)	−0.0011 (3)
C7	0.0164 (5)	0.0137 (4)	0.0132 (4)	−0.0026 (4)	0.0016 (3)	−0.0017 (3)
C8	0.0119 (4)	0.0124 (4)	0.0130 (4)	−0.0014 (3)	0.0001 (3)	−0.0005 (3)

### Geometric parameters (Å, º)

**Table:**

S1—N1	1.6583 (9)	C4—H4	0.9500
S1—C1	1.7248 (11)	C4—C5	1.3896 (14)
S2—C1	1.7363 (11)	C5—H5	0.9500
S2—C2	1.7587 (10)	C5—C6	1.3945 (16)
S3—C1	1.6418 (11)	C6—H6	0.9500
N1—C2	1.2961 (13)	C6—C7	1.3926 (16)
C2—C3	1.4721 (14)	C7—H7	0.9500
C3—C4	1.4024 (14)	C7—C8	1.3910 (15)
C3—C8	1.3981 (14)	C8—H8	0.9500
			
N1—S1—C1	100.79 (5)	C5—C4—H4	120.1
C1—S2—C2	95.54 (5)	C4—C5—H5	119.7
C2—N1—S1	115.28 (8)	C4—C5—C6	120.51 (10)
S1—C1—S2	110.12 (6)	C6—C5—H5	119.7
S3—C1—S1	124.34 (6)	C5—C6—H6	120.0
S3—C1—S2	125.54 (7)	C7—C6—C5	119.96 (10)
N1—C2—S2	118.25 (8)	C7—C6—H6	120.0
N1—C2—C3	122.66 (9)	C6—C7—H7	120.1
C3—C2—S2	119.09 (8)	C8—C7—C6	119.80 (10)
C4—C3—C2	119.78 (9)	C8—C7—H7	120.1
C8—C3—C2	120.68 (9)	C3—C8—H8	119.8
C8—C3—C4	119.54 (9)	C7—C8—C3	120.48 (10)
C3—C4—H4	120.1	C7—C8—H8	119.8
C5—C4—C3	119.70 (10)		
			
S1—N1—C2—S2	0.97 (12)	C2—S2—C1—S1	1.38 (6)
S1—N1—C2—C3	−179.44 (8)	C2—S2—C1—S3	−178.33 (8)
S2—C2—C3—C4	176.91 (8)	C2—C3—C4—C5	−179.15 (9)
S2—C2—C3—C8	−2.91 (13)	C2—C3—C8—C7	178.73 (10)
N1—S1—C1—S2	−1.05 (7)	C3—C4—C5—C6	0.27 (16)
N1—S1—C1—S3	178.66 (7)	C4—C3—C8—C7	−1.09 (15)
N1—C2—C3—C4	−2.67 (15)	C4—C5—C6—C7	−0.80 (16)
N1—C2—C3—C8	177.51 (10)	C5—C6—C7—C8	0.38 (16)
C1—S1—N1—C2	0.08 (9)	C6—C7—C8—C3	0.57 (16)
C1—S2—C2—N1	−1.49 (9)	C8—C3—C4—C5	0.67 (15)
C1—S2—C2—C3	178.91 (8)		

### Hydrogen-bond geometry (Å, º)

**Table:**

*D*—H···*A*	*D*—H	H···*A*	*D*···*A*	*D*—H···*A*
C4—H4···N1^i^	0.95	2.89	3.6281 (14)	136
C5—H5···S1^i^	0.95	3.04	3.8474 (11)	144
C5—H5···S3^ii^	0.95	2.96	3.6529 (11)	131
C6—H6···S3^ii^	0.95	3.11	3.7231 (12)	124
C7—H7···S3^iii^	0.95	3.06	3.9651 (12)	159
C8—H8···S2^iii^	0.95	3.10	3.9141 (11)	145

Symmetry codes: (i) −*x*+2, −*y*, −*z*+1; (ii) *x*+1, *y*, *z*+1; (iii) −*x*, −*y*+1, −*z*+1.
